# A complexity science approach to law and governance

**DOI:** 10.1098/rsta.2023.0166

**Published:** 2024-04-15

**Authors:** Pierpaolo Vivo, Daniel M. Katz, J. B. Ruhl

**Affiliations:** ^1^ Department of Mathematics, King's College London, Strand, London, UK; ^2^ Illinois Institute of Technology Chicago-Kent College of Law, Chicago, IL, USA; ^3^ CodeX, The Stanford Center for Legal Informatics, Stanford, CA, USA; ^4^ Bucerius Law School, Hamburg, Germany; ^5^ Vanderbilt University Law School, Nashville, TN, USA

**Keywords:** complexity, legal complexity, complex systems, law, governance, artificial intelligence

## 1. Introduction

The premise of this Special Issue is that legal systems are complex adaptive systems, and thus complexity science can be usefully applied to improve understanding of how legal systems operate, perform and change over time. The articles that follow take this proposition as a given and act on it using a variety of methods applied to a broad array of legal system attributes and contexts. Yet not too long ago some prominent legal scholars expressed scepticism that this field of study would produce more than broad generalizations, if even that [1–3]. To orient readers unfamiliar with this field and its history, here we offer a brief background on how using complexity science to study legal systems has advanced from claims of ‘pseudoscience’ status [3, p. 8] to a widely adopted mainstream method. We then situate and summarize the articles.

The focus of complexity science is complex adaptive systems (CAS), systems ‘in which large networks of components with no central control and simple rules of operation give rise to complex collective behavior, sophisticated information processing and adaptation via learning or evolution’ [4]. It is important to distinguish CAS from systems that are merely complicated, such as a combustion engine, or complex but non-adaptive, such as a hurricane [4]. A forest or coastal ecosystem, for example, is a complicated network of diverse physical and biological components, which, under no central rules of control, is highly adaptive over time [5].

At hubs of research such as the Santa Fe Institute, complexity science blossomed in the early 1990s and soon took hold in the social sciences, such as economics [6] and sociology [7], and policy analyses including of urban development [8] and national security [9]. Miller & Page [10] broadly grounded the discipline as one that could leverage computational methods to gain deeper understanding of social systems. As an example of how mainstream and ubiquitous complexity science is in the social sciences, following the 2009 global financial collapse, a group of the world's most prominent financial system leaders and researchers argued for greater use of complexity science to inform regulation of financial institutions [11].

Legal systems are social systems—they are composed of networks of institutions (courts, legislatures, agencies) and instruments (laws, regulations, judicial decisions)—and thus it is not surprising that complexity science also included legal systems within its scope. Indeed, in his influential work forging the earliest frameworks of complexity science, Kauffmann [12] posited that common law judicial systems are CAS.

Ruhl & Katz [13] traced the development of complexity science as a method for studying legal systems through three phases. The earliest work in the field was mostly descriptive, aimed at intuitively mapping the features of CAS onto legal systems (e.g. [14,15]). As this scholarship broadened across legal fields it became increasingly prescriptive [16]. Taking it as a given that legal systems are CAS, this work proposed ways to most effectively organize legal institutions and instruments across fields as diverse as bankruptcy law [17], telecommunications law [18] and health law [19].

In fairness to the sceptics, and in contrast to its progress in other social sciences, most of the work framing legal systems as CAS in these descriptive and prescriptive phases was non-empirical. The turning point in this regard came in the late 2000s as legal and policy scholars began applying computational tools, in particular from network science, to explore legal system components and behaviour (reviewed in [13], pp. 216–222), including the growth of legal complexity across various legal systems [20–22]. With publication of this stream of work in prominent peer-reviewed journals, the proposition that legal systems are CAS gained increasing adoption and by 2020 had achieved a substantial degree of acceptance within the mainstream scientific community (e.g. [23–25]).

Since the pioneering works on legal complexity, the vibrant community of legal scholars and practitioners, complexity scientists and artificial intelligence (AI) experts has steadily grown over the years, leading to a much wider range of topics being investigated with a variety of new tools. To name just a few: the study of legal citation networks [26,27], machine-learning and network analysis of statutes, treaties and court litigation [28,29], stat-mech models of judicial decisions [30,31] and of structural complexity of legal texts [32,33], corruption scandals [34], as well as the study of legal language and semantics using quantitative models [35].

As this Special Issue attests, the application of complexity science to study legal systems is now producing a richly diverse and robust body of research. Below we summarize each article, using their respective focus of study to sort them into three groups: (i) legal instruments (e.g. statutes, court decisions, treaties); (ii) legal institutions (e.g. courts, legislatures, agencies); and (iii) legal practice and context (e.g. contract formation; economic effects; ethical implications).

## 2. Legal instruments

Lee & Cantwell [36] develop a minimal model of judicial decision-making that takes into account the judges' individual bias as well as peer interactions in a Court. The model is then benchmarked against the US Supreme Court decisions over the period 1946–2001. The paper aims to model the probability of observing a binary string of *n* votes (+1 or −1) cast by the *n* judges of a Court on a specific case requiring a binary decision, where (i) the judges are subject to a common ‘consensus force’ but may deviate from it at their own individual rates; and (ii) there is a pairwise interaction between judges (and their votes), which is modelled as Ising-like. The model parameters and biases could be then inferred from data using a Bayesian methodology. Implementing this agenda on the second Rehnquist Court and on the Roberts Court, the authors find strong evidence for the combined role of individual biases and interactions between judges, being able to separate and integrate the two contributions and thus reproduce with nearly perfect accuracy the entire distribution of votes ever cast, as well as all relevant statistics that can be extracted from the data. This work is a testament to the beneficial and insightful contribution to knowledge that may arise from the synergy between parsimonious mathematical modelling and skillful calibration on legal data.

In Coupette *et al*. [37] higher-order network interactions—and their time evolution—are considered for the first time in the legal context. The authors consider legal citation networks—sets of judicial decisions that are linked because one cites another in the context of a Court's opinion or decision—and legal collaboration networks—formed by considering arbitrators that participated in the same judicial panels. By allowing (i) multiple nodes to participate in an edge; and (ii) considering *snapshot graphs* encoding the time evolution of the (higher-order) connections between elementary entities, the authors first construct a minimal model of the empirical networks above, and then benchmark their model with data extracted from the corpus of decisions of the Federal Constitutional Court of Germany, and from the corpus of collaborations among arbitrators within the World Bank's International Center for Settlement of Investment Disputes. Based on the way their model is constructed, the authors are able to first define generalized notions of the classical pairwise-network concepts of *centralities*, *motifs* and *communities*, and then hunt for these in their data. The introduction of higher-order interactions as well as of a temporal dimension proves to be very insightful and potentially generalizable to a much wider range of settings and problems in the legal domain, as the authors convincingly argue in their conclusions.

Soh [38] develops a novel automated pipeline for discovering significant topics from legal decision texts. The multi-step method involves the use of penalized regressions and post-selection significance tests. Soh then evaluates the method on two datasets: one involving domain name disputes and another focused upon European Court of Human Rights violation cases. Soh demonstrates that this method is well tailored to both of these otherwise disparate legal contexts. Namely, the method is to identify topics that are qualitatively consistent with legal doctrines in both areas. Overall, this work contributes to a broader literature on automated topic identification, which is an important task in the broader field of legal informatics.

## 3. Legal institutions

Mastrandrea *et al*. [39] apply complex networks methods and tools to analyse the coalitions formed by EU nations and institutions during litigation proceedings at the European Court of Justice over the period 1977–2018. This is a novel application of network theory, where two directed and weighted networks (`Friends' and ‘Foes') are constructed, with nodes representing countries or EU institutions involved in a case either as plaintiff/defendant or as intervening third party, and an edge is drawn between two nodes if they are on the same (Friends) or opposite (Foes) side of the case, with potentially interesting implications and repercussions on foreign policy. Among the most interesting findings (i) Friends and Foes networks display a *disassortative* behaviour—the tendency for nodes to connect with dissimilar nodes rather than similar ones—suggesting that countries and institutions involved in a high number of lawsuits tend to be connected with countries and institutions less active in the litigation process. (ii) Strong correlations among centrality measures suggest that certain member states and institutions hold a prominent role in litigation as source and target of interventions and in bridging the networks' communities. (iii) The modularity of networks points to alignments along regional lines and divisions between EU institutions and member states consistently with previous results from social science research on European integration. (iv) There is a greater degree of reciprocity within the Foes network compared with the Friends network, suggesting a higher level of mutual opposition and conflict among nodes in the Foes network. Although exploratory, this paper provides interesting insights into the functioning of European states and institutions and their foreign policy that can be captured and analysed using sophisticated tools from Network Theory.

Adipudi & Kim [40] develop a conceptual framework for analysing international institutions as complex systems. This framework integrates three distinct areas of study on three different scales: institutional effectiveness, institutional interlinkages and institutional networks. This framework advances the field as there is not an approach within the literature that currently addresses the interdependencies created by an extensive web of relationships as well as the feedback within individual institutions and across many international institutions. The authors illustrate the utility of their approach by exploring a network of 378 multilateral environmental agreements with 810 known issue linkages.

Herron *et al*. [41] use a dynamic influence model to examine the role of the US Supreme Court in influencing the direction of legal discourse in the lower federal courts. Law changes over time in response not only to new technologies or social relations giving rise to novel classes of legal disputes, but also through what the authors describe as ‘discursive shifts’ in how judges discuss the facts and the law in the cases before them. They hypothesize two mechanisms for how an apex court such as the US Supreme Court can subtly influence innovation in legal language in this manner: (1) a selection mechanism where the Court's influence primarily derives from the cases it grants for review under its discretionary jurisdiction, thus identifying the more ‘fit’ innovation among innovations made first in the lower courts and inducing lower courts later to ‘reproduce’ the innovation; and (2) an authorship mechanism in which the Court's influence derives directly from discourse innovations made first in its own opinions, thus inducing lower courts to adopt their superior's innovative language and framing. Building on prior work on topic models, dynamic topic modelling and influence modelling, the authors propose to measure innovation as changes in the distribution of the words associated with a given subject matter over time. Applying this model to the corpus of published judicial opinions in the United States in the period 1975–2000, the authors find that cases selected by the Supreme Court for discretionary review have substantially more innovative language than average appellate court cases. Also, among Supreme Court cases, those that were taken up under the Court's mandatory jurisdiction were not disproportionately innovative. They conclude that the Supreme Court's discursive influence is more substantially attributable to selection, with authorship playing a measurable but secondary role.

Ash *et al.* [42] explore the potential relationship between legal code complexity and population size in US localities. In other words, does the complexity of a municipal code scale with the size of a given city? To evaluate this question, the authors analyse municipal codes from 3259 cities. Various measures of legal complexity are explored including metrics like words, bytes and compressed bytes. The authors identify a positive correlation between the quantity of legal rules within a jurisdiction and the population size of that jurisdiction. Specifically, there is a geometric scaling relationship between legal complexity and jurisdiction population, with a scaling parameter of around 0.2. What is the underlying mechanism driving this phenomenon? The authors suggest that the growth in the law is driven by the need to regulate an increasing number of social interactions between individuals. As the population increases, the number of interactions between individuals also increases in turn leading to a greater need for legal rules and regulations.

## 4. Legal practice and context

Nay *et al*. [43] test the legal analysis abilities of Large Language Models (LLMs) (from smaller and weaker models up to the state-of-the-art, notably OpenAI's GPT-4) in applying US tax law. Performing several experiments and prompting enhancement on different releases of the OpenAI LLM software, the authors are able to conclude that LLMs can already perform at high levels of accuracy even though not yet at expert tax lawyer levels. A human expert in tax law would combine precise knowledge of legal sources and precedents with reasoning, logical and mathematical capabilities to provide professional (relevant and correct) legal advice for any specific and concrete scenario. To test how the LLM algorithms would perform, the authors generate multiple-choice scenarios, consisting of a question—featuring randomly generated facts and figures—and a set of potential answers, only one of which is correct. The problems so generated are brand new, and cannot have been part of any training set of the algorithms tested. The experiments are conducted with different levels of contextual information provided, from no additional legal context provided, up to feeding the exact tax provision that is relevant and dispositive of the case presented. This paper provides strong support to the view that LLMs already have or will shortly reach the necessary capability level to be able to ‘understand’ and apply the law to concrete cases, with profound implications and possible disruption on the future of the legal profession, as well as raising a number of ethical issues (how can we ensure that AI answers are aligned with the law? Can AI be prompted to suggest ways to circumvent the law? And many others).

Goodenough & Carlson [44] observe that as the complexity of society has increased, so has the complexity of law, to a point where we are pushing the effective limits of traditional systems of word-based legal rules. They argue that computational techniques hold the potential to significantly enhance our capacity to express and manage legal complexity, by restating public and private legal rules in computable form amenable to automation. The authors compare two approaches: (i) what they call a ‘words first’ approach starts with the words of traditional legal specification and seeks to directly automate and encode them into executable form via human programming or via some kind of machine learning approach, such as a LLM; or (ii) a ‘code first’ approach that moves directly from an understanding of the behaviour sought by the legal instrument and how it is supported by chains of event and consequence, to then create direct, executable representations of rules supporting such behaviour in the language of code—i.e. skipping the representation of events and consequences first through natural language-based formulations of laws, regulations and contracts. Examining different kinds of transaction and regulatory use cases, they conclude that the code-first approach is best suited when ‘trigger’ or ‘boundary’ conditions are important to performance of the instrument, such as insurance or a non-disclosure agreement, where the dispositive question is whether the necessary elements for triggering an affirmative outcome have occurred or whether there has been some violation of a boundary condition. Similarly, they conclude the same for regulatory settings in which set conditions and standards must be met, such as in municipal building codes. Although word-first approaches may be the only practical options when working with an existing corpus of legacy documents (e.g. thousands of commercial leases), the authors argue that a code-first approach, intelligently developed and deployed, holds the greater promise going forward for managing legal complexity through effective legal automation.

Sichelman & Smith [45] construct a basic toy model of real property relations, and define (and measure) the level of ‘legal modularity’ of the corresponding network model. The paper—aimed at legal scholars and practitioners—maps the classical Hohfeld's theory of ‘fundamental legal relations' into a network model encoding the dense interconnections of legal relations between legal actors. This paper is the first to conceptualize and define networks of legal relations between legal actors, and to apply methods and tools of network theory (notably, the calculation of network modularity) as a proxy to determine clusters of actor pairs that share particular types or degrees of legal relations. The formalism and framework designed in this paper has the potential to address how law guides and is shaped by human behaviour in a more quantitative way, possibly encompassing different areas of law, temporal considerations and higher-order interactions.

Gray *et al*. [46] consider the question to what extent GPT Family Models could assist human annotators in identifying legally relevant factors in a given case. They focus on DIAS (Drug Interdiction Auto Stop) cases in the USA, where police officers—who have the power to stop any driver who violates any of the myriad regulations governing vehicles—are also permitted to detain the vehicle for a sniff by a drug dog if suspicious circumstances are observed suggesting drug possession or trafficking. These circumstances must constitute ‘reasonable suspicion’ to believe that drugs are present, with officers legally required to point to specific observations (factors) that caused them to believe drug trafficking was afoot. The paper describes experiments where paid law students were asked to manually annotate court opinions describing legally relevant factors in a corpus of 211 DIAS cases. Annotating a single case required students to read the entire case, identify what factors courts identified as relevant to the decision, and then proceed to annotate the opinion. Final outputs were cross-checked and quality-checked by a legal expert to ensure consistency and alignment with law and guidelines, and were then used as the gold standards for training data and for evaluating performance on the test set. The authors then train and test fine-tuned LLMs to automate the annotation process and to retrieve relevant legal factors in case decisions. This paper therefore provides a methodological framework that could reduce legal complexity and cost in situations where legal factor analysis is important by employing LLMs to assist and complement human annotation.

Katz *et al*. [47] test GPT-4 and its earlier progenitors on the three components of the bar exam, which in many US jurisdictions must be completed by a legally trained individual to be able to practise law. This paper displays a quite sophisticated technical content and underlying methodology, including for instance a ‘contamination check’ directly assisted by the OpenAI creators to make sure that the exam questions had not been presented before to GPT-4 during its training phase. Although the authors confine themselves to arguably the simplest setting and minimal prompting strategy, the results are already staggering, and decisively point towards the ability of LLMs to already pass the bar exam(s) across the board, and quite comfortably! Amusingly enough, GPT-4 really mimics the exam performance of a good but perhaps not stellar student, with some errors still persisting, especially in areas of law (like the Rule Against Perpetuities) that are widely considered among the most difficult to grasp and interpret correctly even for more seasoned practitioners: in some sense, GPT-4 fails where even good students would fail, but does exceedingly well otherwise. While a word of caution is in order, since LLMs may still hallucinate sources, incorrectly interpret facts, or fail to follow ethical requirements, and therefore still require robust human oversight, it is clear that these results highlight a transformative path that is likely to very shortly disrupt the way legal knowledge is assessed and transferred.

Hagan [48] explores the integration of AI in the legal sector (particularly the justice sector) and emphasizes the importance of prioritizing community perspectives in AI design and policy-making. The article reviews the current literature on how AI can help or undermine community members' access to the civil justice system and presents findings from structured interviews and design sessions with community members, in which they were asked about whether, how and why they would use AI tools powered by LLMs to respond to specific legal problems. While likely not generalizable to all circumstances, the results do highlight a range of future research directions that should be pursued in subsequent work.

Yoon *et al.* [49] explore the potential of AI to help reduce disputes. The authors challenge the optimistic view that AI can significantly improve litigation outcomes. While some argue that AI can enhance efficiency and fairness by accurately predicting case outcomes, the authors contend that the existing literature overlooks the multifaceted nature of litigated disputes. The authors identify three types of disagreements in litigation: disputes over the facts of the case, disputes which turn on applicable rules and disputes regarding how particular rules apply to the given facts. In some of these instances, AI is likely to be better positioned for success than in others. Namely, the authors argue AI is less likely to be successful in the disputes over facts or the underlying applicable rules than in instances where the question of how a particular law applies to an agreed upon set of facts is at issue. Overall, the authors counsel a degree of caution regarding the broad applicability of AI-based systems to litigation and dispute resolution.

## Data Availability

This article has no additional data.
